# Stem Cell Growth Factor-β as a Predictive Biomarker of Response to Chemotherapy and Prognosis in Patients with Advanced-Stage Hepatocellular Carcinoma: A Retrospective Study

**DOI:** 10.3390/cancers16020320

**Published:** 2024-01-11

**Authors:** Masamichi Kimura, Koji Nishikawa, Jun Imamura, Kiminori Kimura

**Affiliations:** Department of Hepatology, Tokyo Metropolitan Cancer and Infectious Diseases Center, Komagome Hospital, Tokyo 113-8677, Japan; kouji_nishikawa@tmhp.jp (K.N.); jun_imamura@tmhp.jp (J.I.); kiminori_kimura@tmhp.jp (K.K.)

**Keywords:** hepatocellular carcinoma, atezolizumab, bevacizumab, stem cell growth factor

## Abstract

**Simple Summary:**

Hepatocellular carcinoma (HCC) is one of the leading causes of cancer-related death, and identifying effective treatments is crucial. In this study, we focused on patients with advanced liver cancer and explored how a specific protein in the blood, i.e., stem cell growth factor beta (SCGF-β), could indicate how well patients would respond to a common cancer drug combination. By analyzing blood samples from patients with HCC before and after treatment, we discovered that high SCGF-β levels predicted poorer outcomes. Accordingly, our research suggests that measuring SCGF-β levels could help physicians better understand patient prognosis and tailor treatment plans more effectively. The insights from our study could lead to more personalized and successful treatment strategies for patients with HCC.

**Abstract:**

In this retrospective study, we investigated the potential application of serum stem cell growth factor beta (SCGF-β) as a biomarker for predicting the therapeutic response and prognosis in patients with hepatocellular carcinoma (HCC) undergoing atezolizumab and bevacizumab (Atz/Bev) combination therapy. Pre- and post-treatment serum SCGF-β levels were measured and analyzed in relation to treatment outcomes and overall survival (OS). Pretreatment SCGF-β levels were associated with treatment response. Patients with SCGF-β levels exceeding the 163,295 pg/mL cutoff experienced significantly reduced OS, with a median OS of 12.03 months, compared to 28.87 months in those with SCGF-β levels at or below this threshold. These findings suggest that SCGF-β can serve as a predictive marker for clinical outcomes in HCC treatment, highlighting the need for prospective studies to further validate these results and clarify the mechanisms underlying SCGF-β-related therapeutic resistance.

## 1. Introduction

Liver malignancies are among the most prevalent forms of cancer and the second most common cause of cancer-related deaths globally [1]. The prognosis of patients with liver cancer is generally unfavorable [2]. Hepatocellular carcinoma (HCC), often occurring in individuals with cirrhosis, is the predominant form of primary liver cancer [3]. The Barcelona Clinic Liver Cancer (BCLC) classification system recommends systemic therapy in patients with intermediate (B) and advanced (C) stages of HCC, especially in cases of widespread disease [4]. Currently, frontline treatment of HCC involves a combination of atezolizumab (Atz) and bevacizumab (Bev) [5]. Although some patients experience substantial improvement, others fail to respond adequately, leading to less than optimal outcomes following Atz and Bev therapy. Chronic inflammation is known to substantially enhance the risk of HCC development [3]. Factors indicative of inflammation, such as cytokines and growth factors, may serve as markers for diagnosing and establishing HCC prognosis. Although several serum biomarkers have been utilized to predict outcomes in advanced-stage HCC, specific serum indicators that can predict treatment response have been poorly explored and identified [6,7,8].

Stem cell growth factor (SCGF), a protein encoded by the *CLEC11A* gene, belongs to the C-type lectin superfamily and functions as a growth factor for hematopoietic progenitor cells [9]. Elevated levels of SCGF-β, a variant of SCGF, have been detected in circulating tumor cells in breast cancer [10] and are linked to increased aggressiveness and metastatic potential in lung cancers originating from drug-resistant cancer stem cells marked by CD133, exhibiting increased levels of angiogenic and growth factors, including SCGF-β [11]. In HCC, elevated SCGF-β levels correlate with a lack of response to interventions such as radiofrequency ablation (RFA) and transcatheter arterial chemoembolization (TACE) [12]. Nevertheless, the potential applicability of SCGF-β in predicting responses to systemic chemotherapy and prognosis in patients with HCC has not yet been comprehensively clarified.

In the current study, we aimed to detect 48 cytokines and growth factors associated with cancer progression to predict the responses to Atz/Bev therapy and the prognosis of patients with HCC. We posited that SCGF-β level may serve as a predictive marker for therapeutic response and prognosis in HCC treatment.

## 2. Materials and Methods

### 2.1. Ethics Statement

The study was approved by the ethics committee of the Tokyo Metropolitan Komagome Hospital (No. 2516). This study adhered to the principles of the Declaration of Helsinki.

### 2.2. Patient Selection and Classification

This retrospective study included data from patients who received the Atz/Bev combination therapy at our institution between October 2020 and November 2023. Inclusion in the study was contingent upon the availability of initial response evaluations following therapy initiation. HCC diagnosis was established through standard radiological assessments using computed tomography (CT) and magnetic resonance imaging (MRI), which revealed characteristic imaging features.

Two months after treatment initiation, the patients were stratified into three groups according to the Response Evaluation Criteria in Solid Tumors (RECIST) guidelines: those who achieved a partial response (PR) (*n* = 9), exhibiting tumor reduction post-treatment; those with stable disease (SD) (*n* = 12), where no change in tumor size was observed; and those with progressive disease (PD) (*n* = 13), where the tumor size increased. Additionally, patients were characterized by sex, age, etiology (including the hepatitis C virus (HCV), the hepatitis B virus (HBV), alcohol consumption, non-alcoholic steatohepatitis (NASH), idiopathic portal hypertension (IPH), or unknown origins), laboratory metrics (such as alpha-fetoprotein (AFP), alanine aminotransferase (ALT), and aspartate aminotransferase (AST)), and disease classification (Child-Turcotte-Pugh (CTP) classification and Albumin-Bilirubin (ALBI) grade).

### 2.3. Quantification of Serum Cytokines

Serum specimens were collected immediately before commencing therapy and at the point of the first evaluative CT scan, 2 months after treatment initiation. These specimens were preserved at a temperature of −80 °C until analysis. Once thawed, serum samples were utilized to quantify the concentrations of 48 different cytokines, chemokines, and growth factors. We employed a premixed Bio-Plex Pro Human Cytokine Screening 48-Plex Panel (Catalog No. 12007283; Bio-Rad Laboratories, Hercules, CA, USA) and performed the assays using the Bio-Plex 200 System (Bio-Rad), strictly adhering to the guidelines provided by the manufacturer.

Herein, we assessed the levels of various cytokines, which included interleukin (IL)-1α, IL-2 receptor alpha (IL-2Ra), IL-3, IL-12p40, IL-16, IL-18, interferon (IFN)-α2, leukemia inhibitory factor, migration inhibitory factor, stem cell factor (SCF), tumor necrosis factor (TNF)-β, TNF-related apoptosis-inducing ligand, along with IL-1β, IL-1Ra, IL-2 through IL-6, IL-9 through IL-17, IFN-γ, and TNF-α. We also measured an array of chemokines, such as cutaneous T-cell-attracting chemokine, growth-regulated oncogene-α, MCP-3, monokine induced by gamma interferon (MIG), stromal cell-derived factor (SDF)-1α, IL-8, eotaxin, monocyte chemoattractant protein (MCP)-1, macrophage inflammatory protein (MIP)-1α, interferon gamma-induced protein 10 (IP-10), MIP-1β, and RANTES. Growth factors that were quantified included hepatocyte growth factor (HGF), macrophage colony-stimulating factor (M-CSF), nerve growth factor-β, SCGF-β, IL-7, basic fibroblast growth factor (FGF), granulocyte colony-stimulating factor (G-CSF), platelet-derived growth factor (PDGF)-B, vascular endothelial growth factor (VEGF), and granulocyte-macrophage colony-stimulating factor (GM-CSF). All cytokines, chemokines, and growth factors analyzed in this investigation are widely known to exert functions in the oncogenesis, progression, and spread of cancer.

### 2.4. Therapy Response Analysis

The revised RECIST guidelines (version 1.1) were used to evaluate treatment-related responses [13]. Treatment response was defined using RECIST-based findings on the first CT scan after initiating Atz/Bev combination therapy.

### 2.5. Survival Analysis

Overall survival (OS) was defined as the duration from the initiation of treatment for HCC until the occurrence of death. During the evaluation, the OS values of patients with low versus high serum SCGF-β levels were compared using Kaplan–Meier survival plots and assessed through log-rank testing. Threshold values were determined by receiver operating characteristic (ROC) curve analysis, which plots the sensitivity (true positive rate) against the false positive rate (100-specificity).

### 2.6. Statistical Analysis

Data analysis was conducted using GraphPad Prism software, version 9.0 (GraphPad Software, LLC, Boston, MA, USA). Continuous variables are presented as mean values with standard deviation (SD) or as medians with interquartile range (IQR). To compare these variables between the two treatment groups, Student’s t-test was applied for parametric data, while the Mann–Whitney U-test was used for nonparametric data. Welch’s ANOVA was utilized to compare three groups. Categorical variables are expressed as percentages, and differences between groups were determined using Pearson’s chi-squared test or Fisher’s exact test, depending on suitability. The Wilcoxon paired signed-rank test was used to assess changes in continuous variables over time from the initial measurement. A *p*-value of <0.05 was considered statistically significant.

## 3. Results

### 3.1. Serum Cytokine and Chemokine Levels in Patients with HCC

**Figure 1:**
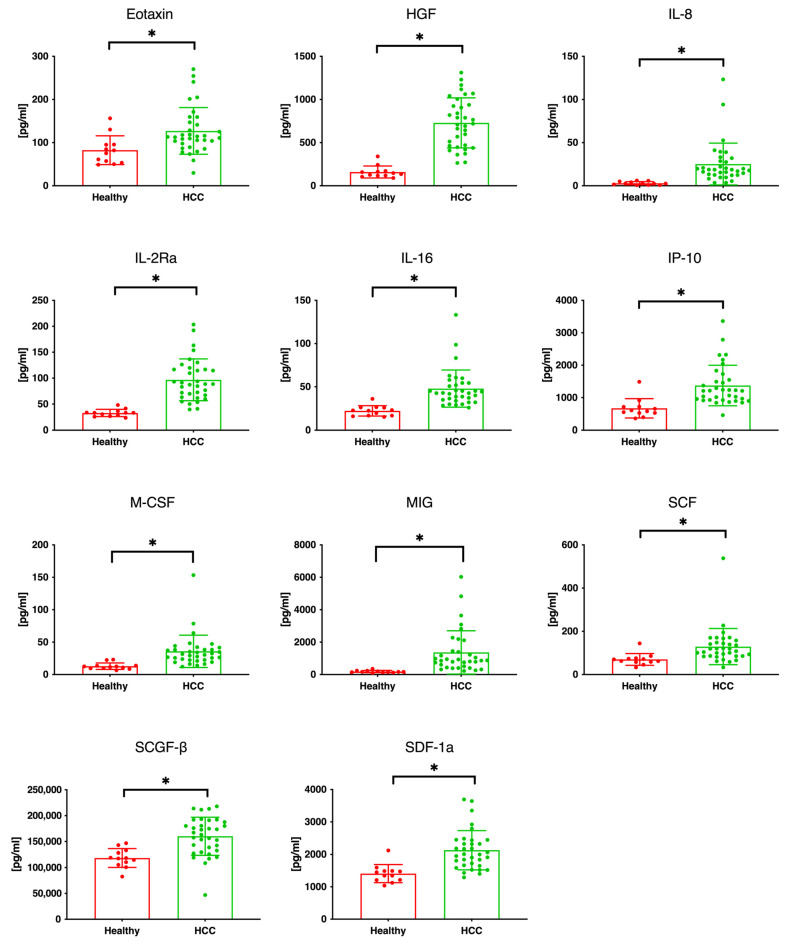
Serum levels of selected cytokines, chemokines, and growth factors in patients with HCC (*n* = 34) and healthy volunteers (*n* = 12). The figure illustrates the comparison of serum levels between these two groups for 11 of 48 measured factors. Specifically, eotaxin, HGF, IL-2Ra, IL-8, IL-16, IP-10, M-CSF, MIG, SCF, SCGF-β, and SDF-1α show statistically significant increases in patients with HCC than in healthy volunteers. HCC, hepatocellular carcinoma; HGF, hepatocyte growth factor; IL, interleukin; 2Ra, 2 receptor subunit alpha; IP, interferon gamma-induced protein; M-CSF, macrophage colony-stimulating factor; MCP, monocyte chemoattractant protein; MIG, monokine induced by gamma; SCF, stem cell factor; SCGF-β, stem cell growth factor-β; SDF, stromal cell-derived factor. In the figure, an asterisk (*) indicates that the *p*-value is less than 0.05, denoting statistical significance.

Table 1 summarizes the demographic and clinical profiles of the patients included in this study. We analyzed 68 serum specimens collected before and after treatment from 34 patients with HCC, comprising 25 men and nine women, with an average age of 69 years (range: 53–83 years). The etiology of HCC was attributed to HCV in 11 patients, HBV in six, alcohol consumption in eight, NASH in three, IPH in one, and undetermined in five patients. Of these patients, 31 were classified as having CTP class A cirrhosis, and 11, 11, and 12 patients had ALBI grades 1, 2a, and 2b, respectively. The maximum tumor diameter measured 4.5 cm (range: 1.1–15.6 cm); the number of tumors ranged from 2 to 13 with a median of 4; and seven of 27 patients were classified as having BCLC stage B/C. Microvascular invasion was observed in seven patients, whereas extrahepatic metastases were identified in 19 patients. The levels of serum AFP and the protein induced by the absence of vitamin K or antagonist-II (PIVKA-II) were 17,230.5 ± 64,448.4 ng/mL and 8041.8 ± 15,280.0 mAU/mL, respectively, with no marked differences observed between the groups showing PD, SD, and PR. Additionally, there were no significant differences in the complete blood count, biochemical, or coagulation test results among the three response groups. Initially, 48 different cytokines, chemokines, and growth factors were quantified in serum samples from 12 healthy volunteers without liver disease and 34 patients with HCC. Before initiating Atz/Bev combination therapy, patients with HCC exhibited significantly elevated serum levels of eotaxin, HGF, IL-2Ra, IL-8, IL-16, IP-10, macrophage colony-stimulating factor (M-CSF), MIG, SCF, SCGF-β, and stromal cell-derived factor 1 alpha (SDF-1α) compared with the healthy volunteers, as depicted in Figure 1.

### 3.2. Serum SCGF-β Levels and Treatment Response Prediction

**Figure 2:**
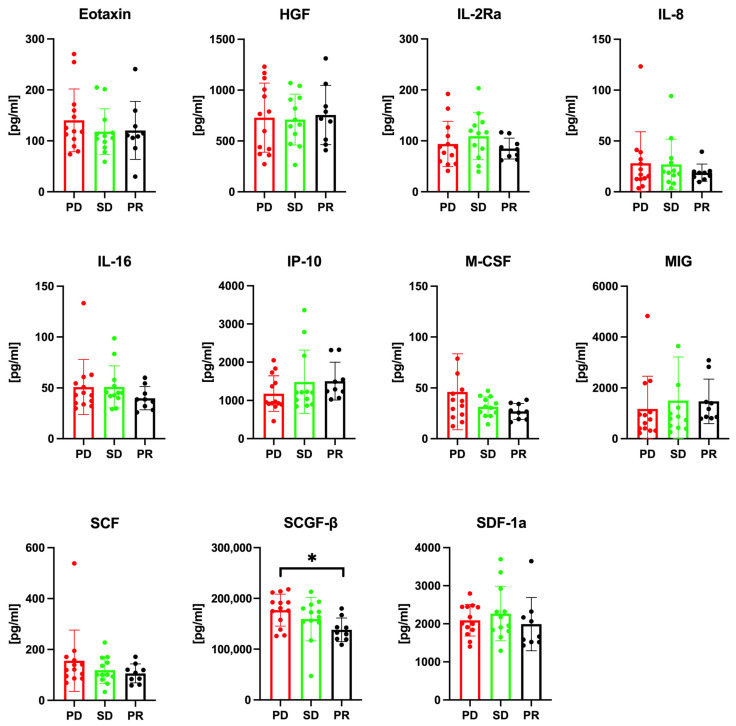
Pretreatment levels of cytokines/chemokines/growth factors between the PR, SD, and PD groups. Serum levels of cytokines, chemokines, and growth factors, including eotaxin, HGF, IL-2Ra, IL-8, IL-16, IL-16, IP-10, M-CSF, MCP-1, MIG, SCF, SCGF-β, and SDF-1α, were measured in samples of the PR (*n* = 10), SD (*n* = 12), and PD (*n* = 12) groups. The PD group exhibits significantly higher serum SCGF-β levels than the PR group. PD, progressive disease; PR, partial response; SD, stable disease. In the figure, an asterisk (*) indicates that the *p*-value is less than 0.05, denoting statistical significance.

**Figure 3:**
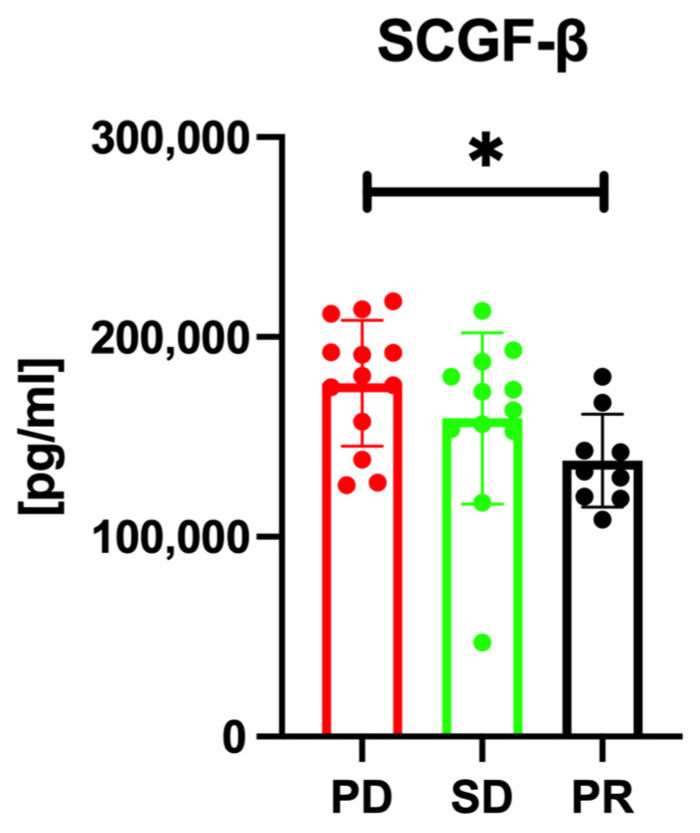
Post-treatment serum SCGF-β levels between the PR, SD, and PD groups. Similar to pretreatment levels, the PD group exhibits significantly higher serum SCGF-β levels than the PR group. In the figure, an asterisk (*) indicates that the *p*-value is less than 0.05, denoting statistical significance.

Subsequent to our initial analysis, we assessed the association between the levels of cytokines/chemokines/growth factors and the treatment response. Pearson’s correlation analysis was conducted across the PR, SD, and PD groups before treatment initiation. The analysis revealed a notable distinction in SCGF-β levels; the PD group displayed significantly elevated SCGF-β levels before treatment when compared with the PR group (*p* = 0.045), as illustrated in Figure 2. The median pretreatment SCGF-β levels were 176,742 ± 31,453 pg/mL in the PD group, 159,282 ± 42,823 pg/mL in the SD group, and 138,097 ± 23,187 pg/mL in the PR group. Likewise, post-treatment SCGF-β levels differed significantly among the groups, with the PD group showing considerably higher SCGF-β levels after treatment than the PR group (*p* = 0.035), as shown in Figure 3. Following treatment, the median SCGF-β levels were 190,078 ± 31,150 pg/mL in the PD group, 162,418 ± 41,735 pg/mL in the SD group, and 151,230 ± 29,677 pg/mL in the PR group. These findings indicate that pretreatment serum SCGF-β levels may predict the response to chemotherapy, and post-treatment levels could be valuable in monitoring the response to Atz/Bev therapy. Additionally, our univariate analysis, as outlined in Table 2, revealed significant associations between elevated serum SCGF-β levels and various tumor and clinical characteristics. This offers initial insights into the role of SCGF-β in the clinical context of HCC. Subsequent to the univariate analysis, a multivariate analysis was performed to ascertain the independent prognostic significance of SCGF-β levels in terms of treatment response and OS, with the outcomes presented in Table 3.

### 3.3. Predictive Value of Serum SCGF-β Level

**Figure 4:**
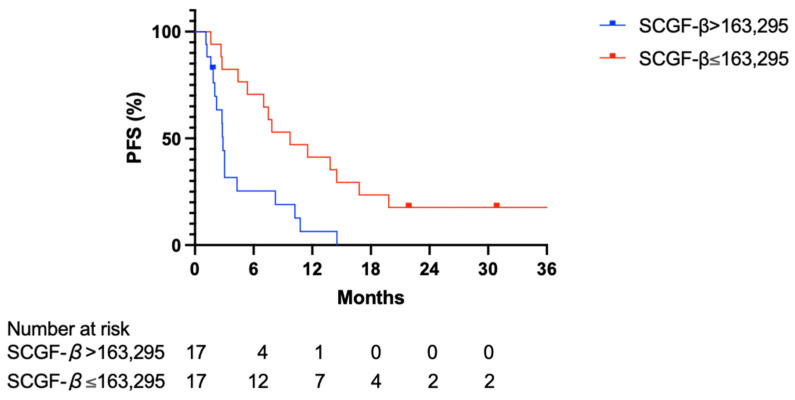

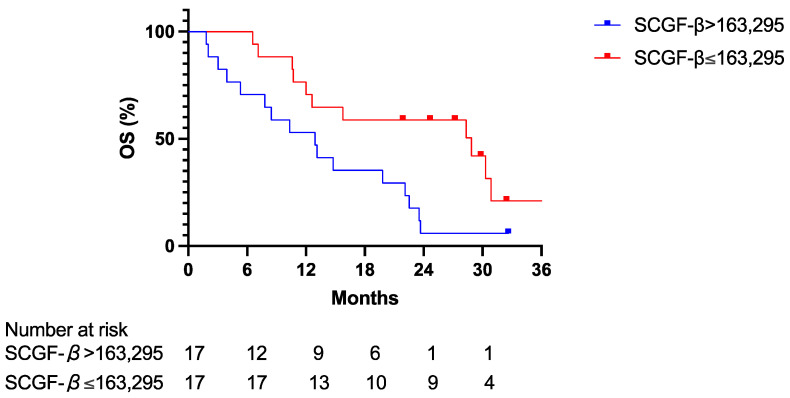
Kaplan–Meier survival curves for PFS and OS relative to SCGF-β levels in patients with HCC. The blue line represents patients with serum SCGF-β levels at or below the 163,295 pg/mL threshold, while the red line depicts those with levels above the threshold. The top panel illustrates PFS, showing a notable divergence in survival probabilities between the two groups. The bottom panel shows OS, once again demonstrating a significant difference in survival outcomes based on SCGF-β levels. The survival data indicate that higher serum SCGF-β levels are associated with shorter PFS and OS in patients receiving Atz/Bev combination therapy for HCC. HCC, hepatocellular carcinoma; OS, overall survival; PFS, progression-free survival.

In the analysis of progression-free survival (PFS), patients with SCGF-β levels above 163,295 pg/mL demonstrated significantly lower PFS than those with levels at or below this threshold (hazard ratio (HR) 0.370; 95% confidence interval (CI) 0.169–0.809; *p* = 0.003). The median PFS for patients exceeding the 163,295 pg/mL SCGF-β cutoff was 2.87 months, whereas it was 9.73 months for those with levels at or below the cutoff. Furthermore, analyzing OS with a SCGF-β threshold of 163,295 pg/mL, patients with SCGF-β levels above the cutoff had significantly lower OS than patients with SCGF-β levels below the cutoff level (HR 0.448; 95% CI 0.208–0.965; *p* = 0.012), as shown in Figure 4. Specifically, the median OS of patients with SCGF-β levels exceeding the 163,295 pg/mL threshold was 12.03 months, whereas that for patients with SCGF-β levels at or below the 163,295 pg/mL value was 28.87 months. Collectively, these results imply that the serum SCGF-β level may be a promising biomarker for predicting pretreatment response and providing prognostic insight for patients with HCC.

## 4. Discussion

In the current study, we evaluated the serum levels of various cytokines, chemokines, and growth factors to determine their predictive value for the efficacy of Atz/Bev therapy in patients with HCC. We observed that individuals with disease progression had notably higher pretreatment SCGF-β levels than those with PR. This trend continued post-treatment, with the PD cohort maintaining elevated SCGF-β levels when compared with the PR group, indicating the potential of SCGF-β as an indicator for monitoring the effectiveness of Atz/Bev therapy during the course of treatment. Intriguingly, upon assessing PFS and OS using a threshold SCGF-β level of 163,295 pg/mL, a substantial decrease in PFS and OS was detected in patients with SCGF-β levels exceeding 163,295 pg/mL. Thus, serum SCGF-β could be a promising marker for predicting the clinical trajectory of patients with HCC.

A comprehensive univariate analysis, as presented in Table 2, revealed a significant association between SCGF-β levels and patient age, suggesting that age may have a crucial role in SCGF-β expression and its implications in HCC.

Furthermore, a multivariate Cox proportional hazards regression analysis (Table 3) confirmed SCGF-β as an independent prognostic factor for treatment response and OS. This underscores the significance of SCGF-β not only as a biomarker for HCC progression but also as a critical factor in predicting patient outcomes following Atz/Bev therapy. The hazard ratios and *p*-values derived from this analysis provide a robust framework for understanding the complex interplay between SCGF-β levels and various clinical variables, highlighting its potential utility in clinical decision-making and patient management.

In light of recent studies, such as one conducted by Goldschmidt et al. [14], which demonstrated the role of SCGF-β in predicting acute cellular rejections in liver transplants, the potential applications of SCGF-β as a biomarker are being increasingly recognized in various medical contexts. Our findings contribute to this growing body of knowledge by suggesting SCGF-β’s potential as a prognostic marker, specifically in the treatment of HCC.

The analysis of PFS further supported SCGF-β’s role as a predictive biomarker, where patients with levels above a certain threshold had significantly lower PFS. This addition to our findings reinforces the predictive capability of SCGF-β for both treatment response and patient prognosis, providing a more nuanced understanding of its role in the clinical management of HCC.

SCGF, encoded by the *CLEC11A* gene, is part of the C-type lectin superfamily and plays a pivotal role in the expansion of early hematopoietic progenitor cells, as produced by bone and hematopoietic stromal cells [9,15]. SCGF is also crucial for the proliferation of leukemic cells via autocrine or paracrine loops, underscoring the importance of SCGF in hematopoietic stem/progenitor cell regulation [16]. Moreover, SCGF has been implicated in the pathogenesis and prognosis of various cancers, including HCC, lymphoblastic leukemia, and testicular germ cell tumors, often correlating with recurrence and diminished OS [17,18,19].

The significance of SCGF-β in cancer evolution and response to therapy has been the focus of research across different cancer types [10,11]. High serum SCGF-β levels have been linked to poor response to treatments like RFA and TACE in HCC, with similar observations documented in the context of breast and lung cancer [10,11,12]. However, the relationship between systemic chemotherapy outcomes and prognostic predictions based on SCGF-β levels in patients with HCC is under investigation.

The precise mechanism through which elevated SCGF-β levels impact the lack of response to Atz/Bev therapy and the subsequent prognosis of patients with HCC is yet to be comprehensively clarified. Atz, a checkpoint inhibitor targeting PD-L1, mediates its function by enhancing T-cell-mediated immune responses to cancer cells [20], while Bev targets VEGF to curb angiogenesis and tumor expansion [21]. It is postulated that the presence of high SCGF-β may disrupt the actions of these drugs, with SCGF-β potentially modifying immune cell operations, thus impacting the therapeutic potency of Atz [22] and possibly promoting angiogenesis, which would mitigate the Bev-mediated effects [23].

The retrospective nature of this investigation and the limited number of participants constitute substantial limitations of this study. To confirm the findings and delve into the mechanisms connecting SCGF-β levels with the response to Atz/Bev therapy in HCC, further studies with larger cohorts and a prospective design are essential.

## 5. Conclusions

The findings of the current retrospective study suggest that the pretreatment serum level of SCGF-β could be a significant predictor of therapeutic response and prognosis in patients with HCCs undergoing Atz/Bev combination therapy. Patients with SCGF-β levels above the threshold of 163,295 pg/mL not only exhibited a reduced median PFS of 2.87 months, compared to 9.73 months for those with lower levels, but also a median OS of 12.03 months, in contrast to 28.87 months for those below the threshold. Taken together, these findings underline the potential of SCGF-β not only as a biomarker for assessing the efficacy of Atz/Bev therapy but also as a prognostic indicator for clinical outcomes. Given that the association between high SCGF-β levels and non-responsiveness, as well as its potential for targeting in HCC treatment, are yet to be clarified comprehensively, future studies are essential. Prospective studies with larger cohorts are particularly needed to elucidate the mechanisms of SCGF-β-related therapeutic resistance and to refine personalized treatment strategies for HCC, potentially improving patient management and survival outcomes.

## Figures and Tables

**Table 1:** Patient background and clinical characteristics.

	PD	SD	PR	Total	*p*-Value
Patient characteristics					
Male/Female	11/1	6/6	8/2	25/9	ns
Age (years)	64 ± 10	71 ± 13	74 ± 6	69 ± 11	ns
Etiologies	12	12	9	34	
HBV	4	2	0	6	ns
HCV	4	3	4	11	ns
Alcohol	3	4	1	8	ns
NASH	0	1	2	3	ns
IPH	0	1	0	1	ns
Unknown	1	1	3	5	ns
Clinical characteristics					
CTP class A/B	10/2	11/1	10/0	31/3	ns
ALBI grade 1/2a/2b	6/2/4	2/6/4	3/3/4	11/11/12	ns
Maximum tumor diameter, cm, median (range)	4.0 (1.1–15.6)	4.8 (1.5–13.5)	5.0 (1.8–10.2)	4.5 (1.1–15.6)	ns
Number of tumors, median (range)	4 (2–9)	4 (2–13)	4 (2–10)	4 (2–13)	ns
BCLC stage B/C	2/11	3/9	2/7	7/27	ns
MVI	2	3	2	7	ns
Extra-hepatic metastases	10	7	2	19	ns
γ-GTP (IU/L)	191 ± 177.1	142.2 ± 168.1	160.0 ± 177.2	165.5 ± 175.3	ns
Albumin (g/dL)	3.8 ± 0.5	3.6 ± 0.3	3.2 ± 0.4	3.7 ± 0.4	ns
T-Bil (mg/dL)	0.96 ± 0.80	0.95 ± 0.50	0.83 ± 0.38	0.92 ± 0.62	ns
Sodium (mmol/L)	140.6 ± 1.8	139.8 ± 2.0	139.3 ± 4.4	140.0 ± 2.8	ns
Cre (mg/dL)	0.85 ± 0.16	0.78 ± 0.19	0.81 ± 0.16	0.81 ± 0.18	ns
WBC (10^3^/μL)	5.9 ± 1.4	5.6 ± 2.0	5.8 ± 2.6	5.8 ± 2.0	ns
Hb (g/dL)	12.7 ± 1.9	12.5 ± 1.8	12.5 ± 1.9	12.6 ± 1.8	ns
Plt (10^4^/μL)	21.1 ± 7.4	14.8 ± 5.9	15.3 ± 6.8	17.3 ± 7.3	ns
PT-INR	1.01 ± 0.1	1.06 ± 0.1	1.03 ± 0.1	1.04 ± 0.1	ns
AFP (ng/mL)	43,499.6 ± 98,693.4	913.7 ± 1632.0	1042 ± 2137.9	17,230.5 ± 64,448.4	ns
PIVKA-II (mAU/mL)	8693.2 ± 15,344.7	9664.7 ± 17,505.5	4937 ± 10,934.0	8041.8 ± 15,280.0	ns

PD, progressive disease; SD, stable disease; PR, partial response; ns, not significant; HBV, hepatitis B virus; HCV, hepatitis C virus; NASH, non-alcoholic steatohepatitis; IPH, idiopathic portal hypertension; CTP, Child-Turcotte-Pugh; ALBI, albumin-bilirubin; AST, aspartate aminotransferase; ALT, alanine aminotransferase; ALP, alkaline phosphatase; γ-GTP, γ-glutamyl transferase; T-Bil, total bilirubin; Cre, creatinine; WBC, white blood cell; Hb, hemoglobin; Plt, platelet; PT-INR, prothrombin time-international normalized ratio; AFP, alpha-fetoprotein; PIVKA-II, protein induced by vitamin K absence or antagonist II.

**Table 2:** Univariate analysis of SCGF-β levels with clinical and tumor characteristics.

Variable	Category/Unit	High SCGF-β Level (*n* = 17)	Low SCGF-β Level (*n* = 17)	*p*-Value	Odds Ratio (OR)	95% CI for OR
Sex	Male vs. Female	14 (82.4%)	11 (64.7%)	0.4384	2.545	0.5473 to 10.66
Maximum tumor diameter	cm	6.8 ± 4.8	10.2 ± 2.9	0.4280	-	-
Number of tumors	Count	5.0 ± 2.7	4.7 ± 3.1	0.4624	-	-
MVI	Present vs. Absent	4 (23.5%)	3 (17.6%)	>0.9999	1.436	0.3289 to 6.498
Extrahepatic metastases	Present vs. Absent	8 (47.1%)	8 (47.1%)	>0.9999	1	0.2629 to 3.803
Age	Years	65.6 ± 10.9	73.0 ± 10.8	0.0219	-	-
Child-Pugh	B vs. A	1 (5.9%)	2 (11.8%)	>0.9999	0.4688	0.0307 to 4.445
AFP	ng/mL	15,706 ± 58,207	18,755 ± 73,712	0.1309	-	-
PIVKA-II	mAU/mL	9197 ± 15,487	6887 ± 15,920	0.4332	-	-

MVI, microvascular invasion; SCGF-β, stem cell growth factor-β.

**Table 3:** Multivariate Cox regression analysis showing the impact of clinical and molecular factors on overall survival in patients with HCC.

Variable	Coefficient (β)	Standard Error	95% Confidence Interval	Hazard Ratio (Exp [β])	*p*-Value
Sex (Male)	0.3309	0.5644	−0.7720 to 1.474	1.392	0.123
Maximum tumor diameter	0.2121	0.077	0.06030 to 0.3664	1.236	0.004
Number of tumors	0.06756	0.08408	−0.1032 to 0.2313	1.07	0.431
Microvascular invasion (MVI [+])	−1.128	0.6639	−2.564 to 0.1000	0.3237	0.081
Extrahepatic metastases ([−])	0.6563	0.6935	−0.6543 to 2.093	1.928	0.324
Age	0.01476	0.02541	−0.03408 to 0.06639	1.015	0.535
SCGF-β [High]	1.563	0.5073	0.6020 to 2.616	4.774	0.002
Child-Pugh [B]	3.632	0.9318	1.756 to 5.516	37.79	<0.001
AFP	9.5 × 10^−6^	4.37 × 10^−6^	5.67 × 10^−7^ to 1.819 × 10^−5^	1	0.047
PIVKA-II	−2.62 × 10^−5^	1.74 × 10^−5^	−6.42 × 10^−5^ to 5.32 × 10^−6^	1	0.101

## Data Availability

The data supporting the findings of this study are available from the corresponding author, Masamichi Kimura, upon reasonable request.
